# Notices

**Published:** 1864-06

**Authors:** 


### NOTICES.

"POPULATION of the United States in 1860, compiled from the original returns of the Eighth Census, under the direction of the Secretary of the Interior, by JOSEPH C. G. KENNEDY, Superintendent of the Census," issued at Washington, from the Government printing-office, 1864. This is a book of 694 pages, eleven inches long and ten broad. On opening it, whole pages of columns of figures strike the eye, as dry as a bone, at the first glance; but on a more minute examination, they are so admirably arranged, so systematic, concise, and full, we at once perceive that the whole volume is rich with information; it affords food for thought and reflection and comparison of the richest and most instructive character. A thinking man might feast on it for a month, and still turn over its leaves with an absorbing interest. It is one thing to string together the number of men, women, children, horses, dogs, and cats of a country; to say how many have died, how many married, how many born, and where they all come from. It is quite another thing to have a mind capable of placing these statements before the reader in such a manner as to make them of the highest interest, and to be a vehicle of instruction at once practical, useful, and of permanent value. For this purpose Congress acted wisely and well in intrusting this important and great work to Mr. Kennedy, who is universally acknowledged to be the most competent man in the nation by all odds for performing the work. In some future number we purpose giving several pages of curiosities of the census; marriage; which live longest, bachelors and maids, or married people? who oftenest remarry, and do so the soonest, widows or widowers? etc.

"Constitution of Nature," by William Andrew, Milwaukee, 1864, paper cover, 8vo, 100 pages. Proposes theories to unfold nature, matter, and vacuum; relative motion and rest; matter the cause of density; vacuum the cause of expansion or porosity; why bodies fall; capillary attraction; combustion; the universe; motion; heat; nature of the planets; weather, life, vital force, etc. These are suggestive themes and have occupied the thoughts of philosophers of all ages, and are treated of by the author in a calm, dignified spirit and with convincing power.

The American Tract Society, Boston, and No. 13 Bible House, New-York, have issued Vol. Ten of the Temperance Tales, by Lucius M. Sargent, and it is not inferior in interest to any of its predecessors. Among the subjects are The Life Preserver, The Prophets, Margaret's Bridal, Temperance Meeting in Tatter-town. Dove Hamilton, or Sunshine and Shadow, printed from the London Religious Tract Society, is a sweet little book of 292 pages, and full of practical household truths; no one can read it, old or young, rich or poor, without deriving rich instruction from it. Among the subjects are: Bread cast upon the Waters, A Mother's Blessing, Going Home, Dove's First Grief, The Surprise, The Stepmother, The New Home, A Labor of Love, Use of Old China, Bread found after many Days, How all came Right at Last.

The American Tract Society, 150 Nassau street, New-York, have issued an interesting little volume, and instructive to the old as well as the young, entitled "The Chosen Friends," or the twelve disciples, being a short biography of each of the twelve disciples of the New Testament. Also "Out of the House of Bondage," and "Friendly Counsels," by Rev. J. B. Waterbury, D.D. Another volume of 235 pages, "Helen Maurice; or, the Daughter at Home," is well worthy of being placed in the hands of every daughter in the land, and is written in a manner calculated to leave a lasting impression as to the duties and responsibilities of life. We hope it may be read and pondered over by many thousands.

The Housekeeper's Guide, and every body's hand-book, containing over five hundred new and valuable recipes and references to household affairs, cooking, with a medical department, mechanic's department, and farmer's department, sixty-four pages; a pamphlet which will be prized by every good housekeeper. Published by Smith and Swinney, chemists, Cincinnati, Ohio, 1864. Price per copy, one dollar. It ought to be sent post-paid to Guinea for fifteen cents.

The Northern Monthly, a magazine of literature, civil and military affairs. Portland, Maine. Edward P. Weston, Editor. $2 a year, single numbers 20 cts.; size and shape of the Atlantic Monthly; 70 pages. Among the contributors to the April No. 2 are Caroline E. D. Howe, Miss S. P. Warren, John

